# “Why involve older people in research?” Revisiting Alan Walker’s earlier editorial based on recent experiences from the UserAge research programme

**DOI:** 10.1186/s40900-023-00493-8

**Published:** 2023-09-11

**Authors:** Sara Hultqvist, Elizabeth Hanson, Håkan Jönson, Björn Slaug, Susanne Iwarsson

**Affiliations:** 1 Department of Health Sciences, Faculty of Medicine, Lund University, P.O. Box 157, 221 00 Lund, Sweden; 2 Department of Health and Caring Sciences, Linnaeus University, 39182 Kalmar, Sweden; 3 School of Social Work, Faculty of Social Sciences, Lund University, 221 00 Lund, Sweden

## Abstract

Posed 16 years ago in a much-cited editorial by gerontologist, Alan Walker, “Why involve older people in research?” is a question that has since inspired researchers in many countries and from diverse disciplines. In Sweden, researchers and older people have been collaborating in the 6-year UserAge research programme, focusing on user involvement in research on ageing and health, UserAge aims at contributing to an in-depth understanding of the challenges and benefits of user involvement in different phases of the research process. Approaching programme completion, the authors take the opportunity to dwell upon current reasons for and modes of user involvement in ageing research in light of the argument originally put forward by Alan Walker back in 2007.

## Walker’s argument

Reflecting on experiences in his role as Director of the English Social Research Council’s Growing Older Programme (1999–2004) in the United Kingdom, Professor Alan Walker posed the question, “Why involve older people in research?”, which has since inspired researchers in many countries and from diverse disciplines. Over the years, Walker has directed numerous major research programmes upholding user involvement as a key principle. In his editorial published in “Age and Ageing” (2007) he pointed to the fact that while some researchers regard older people as subjects or objects of their investigations, nevertheless many researchers in the field of ageing endeavor to do research together with, rather than on or about, older people.

According to Walker, there are two main reasons why older people should be involved in research. One has to do with quality. If researchers want to produce findings that might contribute to the quality of life of older people or the quality of the services and/or products they use, it is essential to involve older people so that they can contribute with their own understandings about ageing and the services and/or products in question. The other reason is linked to human rights:

“[…] as a matter of human rights, like any human research subjects, older people have a right to be consulted about research that is being conducted on them. Arguably this imperative is particularly strong with regard to older people because of their experiences of age discrimination and other forms of social exclusion. The only question, therefore, should be how much consultation/involvement?”

Walker also in his editorial, identified three major historical developments that have given the question of involving older people in research increased impetus. Firstly, the social and cultural transition in post-industrial societies, which entailed changes in the relations between the individual and the state, the most evident manifestations being the rise of consumerism and individualism. The manifestations took shape as a ‘consumer’ perspective aimed at making services more ‘responsive’. Representing a major group of the care and service consumers, older people’s preferences became important. Secondly, as a consumer perspective was increasingly reflected in research policy, researchers had to display an ethos for user involvement in order to secure research funding. Here, Walker concluded that there was a dearth of models of good practice to draw on. Thirdly, this top-down policy driven development ran in parallel with a growth of social movements at grass-roots level representing various groups. The disability movement was a strong force with an agenda aimed at independence and control of their own lives. Walker noted that such self-advocacy had become increasingly relevant for older people and their interest organizations.

Sixteen years after the publication of Walker’s much-cited text, user involvement in ageing research is high on the international research policy agenda, and various approaches for co-research have emerged (e.g., [1, 2]). Aiming to contribute to a more comprehensive understanding of the challenges and benefits of user involvement in different phases of the research process, the ambition of this paper is to revisit Walker’s key arguments in the context of our more recent experiences and findings from a 6-year research programme.

## The UserAge programme

**Fig. 1:**
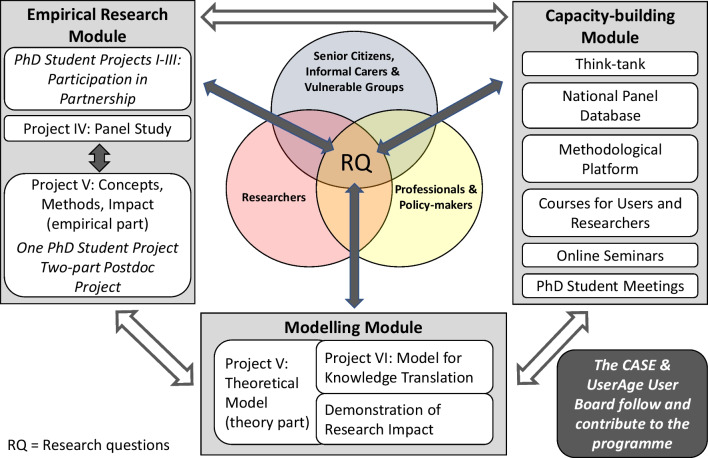
UserAge was implemented as a three-module programme

Implemented by a transdisciplinary team engaging researchers and older people in Sweden, the UserAge program relates to Walker’s question “Is there any evidence that this involvement benefits either research or older people themselves?” [3], p. 482]. UserAge was implemented as a three-module programme (Fig. 1). The empirical module of the programme comprises five PhD student projects, one postdoc project and a panel study. The capacity-building module includes a methodological platform, a think tank on participation, online seminars and PhD student meetings. Including a theorybuilding ambition, the third module focuses on concepts and terminology, existing and emerging theories about user involvement, knowledge translation and generation of impact (for detailed information, see [4]).

In summary, our focus within UserAge is rather on how more than why involve older people in research, drawing on the experiences gained and the lessons learned from our research programme. More specifically, within this correspondence paper we shall:

1. elaborate on *the need to go beyond the heterogenous category of ‘older people’*
2. argue that there is *no need for new models but still urge for more methods to choose among*
3. *caution against tokenism*
4. provide an example of how to *channel a target group’s organized interest*

These four topics are directly related to Walker’s discussion of the historical developments that have contributed to strengthen user involvement in research. We want to add an additional topic that was not touched upon in the editorial, but has nevertheless appeared as an important consideration during the UserAge programme, given its Swedish context. Namely, the issue of *language and terminology*.

### A need to go beyond the heterogenous category of ‘older people’

Should the only question be the extent to which older people should ideally be involved? To move research with and about involvement of older people forward, results from the UserAge programme demonstrate that it is imperative to qualify the definition of different categories of people involved. That is, to specify who among the heterogenous category of older people are we talking about? Frail older people [5], representatives of senior citizens’ interest organizations, and the older segment of the general public [6] each have distinctly different needs of, prerequisites for and attitudes to research involvement. Further, some older people involved in research speak from other positions than those primarily linked to their own chronological old age or mandate given from a certain interest organization. Informal carers are one such example. Informal carers are persons providing care, help or support on a regular basis to someone with whom they have a relationship [7]. Numerous informal carers aged 65 years of age or older can also be categorized as ‘older people’. In Europe, informal carers are predominantly female spouses, daughters and daughters-in-law who are middle-aged or older [8]. In the Swedish studies in the UserAge programme, older people with higher formal education were overrepresented as partners in research. As to gender, we did not observe any differences regarding representation. It is important though to acknowledge that we failed to involve people with other ethnic identities than Swedish to a sufficient extent. This is considered a methodological weakness within our programme. Also, it seems to reflect a general bias, since people from ethnic minority groups are often underrepresented not only in participatory research but also in clinical trials [9]. Questions of roles and representation are always important to address in participatory research. However, we can expect their complexity to increase through the life-course as we tend to collect roles through life-experiences. Therefore, these questions require careful attention when we as researchers want to recruit individuals and groups that are ‘rich in years’.

### No need for new models, but for more methods

While models for user involvement were lacking in 2007, current literature displays a multitude of examples. In their literature review on Patient and Public Involvement (PPI), Greenhalgh et al. [10] identified 65 different models but concluded that few had been used by others than those who developed them. In the UserAge programme we started out with a goal to contribute to model development, but the team of researchers and older people ended up with a decision not to pursue this ambition. The fact that the older people involved in UserAge clearly communicated that such modelling was difficult for them to relate to and rather counteracted than facilitated their involvement is an experience contrasting Greenhalgh et al.’s recommendation to co-create models fitting the local context. Nevertheless, we do see a pressing need for a wider repertoire of methods to choose from when involving a range of older people in different kinds of research. The question “what methods are optimal both with regard to the older people involved and with regard to the issue under study”? should be posed as part of the preparations for each new study. This question requires us to continue developing and trying out appropriate ways of conducting research. We have experienced the need to adapt established methods such as research circles [11] and to also explore more innovative methods such as photo voice [12], to enhance impact and enable the involvement of different groups of older people in research.

### Be aware of tokenism!

The consumer perspective mirrored in research funders’ demand for user involvement, raised by Walker, has become increasingly manifest since 2007. Initiatives such as those emerging during the first decade of the 21st millennium in the UK are now increasingly mainstream at EU level, for example, within the key funding programmes for research and innovation. In Sweden, governmental research councils and private foundations have explicit demands for collaboration between researchers and non-academic partners [13]. That is, provided researchers present adequate research plans for involving older people in their research, funding is available. However, recent findings indicate that we cannot assume that the realization of plans for involvement of older people and other user representatives will take place in a careful, considered or reflective way even if such plans are presented at the proposal stage for funding [14]. These findings are in line with other studies investigating research labeled as ‘participatory’. An obvious risk is that user involvement becomes more of a bureaucratic, scripted activity as opposed to a concrete, genuine strategy well-founded to fit the research aim and questions [15].

### Channeling organized interest

Walker discussed interest groups as an increasingly important force in research on ageing. While the disability movement for decades had been a powerful actor in disability studies, senior citizens’ organizations had only recently entered the scene. An infrastructure to channel the organized interest/s of groups of people within a certain research field can be arranged in different ways. In the UserAge programme a User Board has been engaged. The User Board was set up already in 2010, when established for the Centre for Ageing and Supportive Environments (CASE) at Lund University. Chaired by a representative for a major senior citizens’ organization, the Board consists of a variety of older people bringing with them a wide range of knowledge and experiences. Here, the major senior citizen’s organizations have been key in the recruitment process. Personal networks as well as a pool of people who had shown interest in previous and ongoing research activities at CASE have also been crucial. User Board members have been actively involved in the UserAge programme already from the proposal and research question definition phases. Board members have been involved in designing studies and piloting questionnaires, but also in the strategic planning of the research process as well as in the interpretation of empirical findings. As Sweden has a strong tradition of corporativism, self-advocacy is mainly channeled through established organizations such as the National Organization of Pensioners and Swedish Association for Senior Citizens. Both are represented in our User Board. Thus, some members represent these organizations rather than themselves individually. With the constant challenge to engage a variety of older people, issues of representation are a frequent topic for discussion within our programme.

### The issue of language and terminology

While many of the issues that we have revisited repeatedly during the 6-year UserAge program period can be directly linked to Walker’s argument, there is one theme that has appeared in our Swedish context that was not touched upon—namely the terminology and language issue. The lack of an established terminology to describe collaboration and involvement in research requires specific attention Greenhalgh et al. [10]. We argue that an accepted nomenclature of collaboration, a collaborology, would facilitate knowledge accumulation [16]. Operating in a country where English is not the first language, both the use of scientific terminology in Swedish and scientific communication in English poses challenges and has required constant attention and action in the UserAge programme. Seldom reflected in the scientific literature, there is a balancing act to be considered in terms of communicating in English to reach an international audience and communicating in a national language to make the research accessible and relatable to older people in the local setting. Acknowledging the rationale and need to communicate in both languages depending on the audience and purpose of the communication, we decided to publish papers as well as an anthology in Swedish, along with a report that summarizes the four PhD dissertations and 26 published articles from the programme (e.g., [17–19]). However, while advantageous for knowledge translation in the national context, unless the researchers invest in extensive translation and endeavor to solve issues related to dual publication of original work, the disadvantage is that some results do not reach the international scientific community.

## Concluding remarks: involvement as promotion of democratization and realization of human rights

Though democracy as a concept does not occur in the Walker editorial, in our reading and re-reading, the strive for increased democracy is intertwined into the historical developments put forward by Walker—changes in the relations between the individual and the state, the rise of consumerism aiming at making services more ‘responsive’ to citizens’ needs and a growth of social movements representing various groups. Consequently, democratization has brought to the fore the question of whose knowledge is recognized [20, 21]. Moreover, Walker explicitly referred to human rights as a main reason for promoting the involvement of older people in research and argued that those who are concerned should have a say in issues that concern them. These two reasons could easily be interpreted as strive for democracy.

In the UserAge programme. We have experienced numerous examples of the benefits of user involvement for the research as such as well as for the older people involved. One concrete example is the user forum arranged to develop and optimize both an online survey questionnaire and design for the panel study. This resulted in co-produced methodology, which integrated the perspectives of researchers and older people representing the target population, thus strengthening the validity of the research. As to user benefits, the older people involved in the user forum reported that they learnt a lot and enjoyed the opportunity to get actively involved in methodology development. Given the heterogeneity of the ageing population, we argue that the experiences and findings from UserAge help to nuance and deepen the understanding of user involvement in research. Such knowledge enables researchers to fine tune their methodologies and approaches to better serve specific aims when involving older people in research. While some of our findings relate specifically to the involvement of older people, others are generalizable and valid for individuals of all ages. The risk of tokenism is a general one. The widespread academic habit of not publishing findings in a national language but solely in English, is probably less of a problem in participatory research with youth than with older people.

To conclude, we consider that Walker’s editorial still fuels discussions that need to be open and ongoing within ageing research. A type of context-dependent research work must be applied, which involves a range of methods as well as language. Knowledge of different approaches that are more or less optimal depending on the group involved or the research objectives is essential. Further, innovative methods still need to be developed. Lessons learned—from successes and from failures—need to be systematically documented and evaluated, and critically discussed and shared across disciplines and above all, with partners outside academia. Walker argued that the question should not be *if*, but rather *how much* involvement of older people in research. Let us take this as an invitation for continuous work—adding questions on *who*, *when* and *for what specific purpose*—concerning user involvement of older people in research.

## Data Availability

Data collected in the referred UserAge studies will, after de-identification, be available on reasonable request after publication in peer reviewed journals. The prerequisite for this is a data transfer agreement, approved by legal departments of the institutions of both the requesting researcher and the researchers that provided data for the study. Proposals should be directed to: sara.hultqvist@lnu.se.
